# Polyclonal anti T-lymphocyte antibody therapy monitoring in kidney transplant recipients: comparison of CD3^+^ T cell and total lymphocyte counts

**DOI:** 10.31744/einstein_journal/2018AO4278

**Published:** 2018-11-29

**Authors:** Fabiani Palagi Machado, Alessandra Rosa Vicari, Fábio Spuldaro, João Batista Saldanha de Castro, Roberto Ceratti Manfro

**Affiliations:** 1 Universidade Federal do Rio Grande do Sul, Porto Alegre, RS, Brazil; 2 Hospital de Clínicas de Porto Alegre, Porto Alegre, RS, Brazil

**Keywords:** Kidney transplantation, Immunosuppression, T-lymphocytes, Monitoring, immunologic, Receptor-CD3 complex, antigen, T-cell, Transplante de rim, Imunossupressão; Linfócitos T, Monitorização imunológica, Complexo receptor-CD3 de antígeno de linfócitos T

## Abstract

**Objective:**

To investigate the correlation between total lymphocyte and CD3^+^ T cell counts in peripheral blood in renal transplant patients treated with anti-thymocyte globulin, and discuss related outcomes.

**Methods:**

A single-center, retrospective study involving 226 patients submitted to kidney transplant between 2008 and 2013, and treated with anti-thymocyte globulin for induction or treatment of cellular rejection. Doses were adjusted according to CD3^+^ T cell or total lymphocyte counts in peripheral blood.

**Results:**

A total of 664 paired samples were analyzed. The Spearman's correlation coefficient was 0.416 (p<0.001) for all samples combined; the overall Kappa coefficient was 0.267 (p<0.001). Diagnostic parameters estimated based on total lymphocyte counts were also calculated using the number of CD3^+^ T cells (gold standard), with a cut off of >20 cells/mm^3^.

**Conclusion:**

Total lymphocyte and CD3^+^ T cell counts in peripheral blood are not equivalent monitoring strategies in anti-thymocyte globulin therapy.

## RESUMO

**Objetivo:**

Investigar a correlação entre a contagem de linfócitos totais e células T CD3^+^ no sangue periférico em receptores de transplante renal submetidos a tratamento com globulina antitimocitária, e discutir resultados relacionados.

**Métodos:**

Estudo retrospectivo de centro único envolvendo 226 pacientes submetidos a transplante renal entre 2008 e 2013 e tratados com globulina antitimocitária, para fins de indução ou tratamento de rejeição celular. As doses foram ajustadas de acordo com a contagem de células T CD3^+^ ou linfócitos totais no sangue periférico.

**Resultados:**

No total, 664 amostras pareadas foram analisadas. O coeficiente de correlação de Spearman para as amostras em geral foi de 0,416 (p<0,001) e o coeficiente Kappa, de 0,267 (p<0,001). Os parâmetros diagnósticos estimados com base na contagem de linfócitos totais foram recalculados, empregando-se o número de células T CD3^+^ (padrão-ouro) e adotando-se o ponto de corte >20 células/mm^3^.

**Conclusão:**

A contagem de linfócitos totais no sangue periférico não substitui a contagem de células T CD3^+^ enquanto estratégia de monitorização da terapia à base de globulina antitimocitária.

## INTRODUCTION

Renal transplantation is the current treatment of choice for well selected patients with end-stage kidney disease. Transplantation improves patient quality of life and reduces mortality compared to chronic maintenance dialysis. Over the few last decades, advances in immunosuppressive therapy, better diagnostic and therapeutic methods and improved surgical techniques have led to increased patient survival and long-term graft function, making renal transplantation a cost-effective treatment.^(^ ^1^ ^)^ Deeper understanding of immune responses to allografts revealed that T lymphocytes play a key role in acute cellular rejection.^(^ ^2^ ^)^ Current immunosuppressive therapies are effective in controlling T cell-mediated immune responses. Antibody depletion therapy is widely used in the initial phase of transplant, particularly in patients with high immunological risks. These agents are also employed to treat patients with severe forms of acute rejection.^(^ ^3^ ^)^

In the last two decades, anti-thymocyte globulin (ATG) has been used for acute allograft rejection prophylaxis and treatment,^(^ ^4^ ^)^ and has been associated with significantly reduced rates of delayed graft function, improved early allograft function and shorter hospital stay.^(^ ^5^ ^)^ The ATG is a polyclonal antilymphocyte agent, consisting of a wide variety of T-cell epitope-specific antibodies, which induce rapid and profound CD3^+^ T lymphocyte depletion in peripheral blood.^(^ ^6^ ^)^ In the past, wide use of doses adjusted exclusively for body weight and related adverse effects have often led to over-immunosuppression. Monitoring strategies were then developed to minimize adverse effects and reduce costs while maintaining treatment efficacy. At present, monitoring based on total lymphocyte or CD3^+^ T cell count in peripheral blood is recommended.^(^ ^7^ ^)^ The first strategy is obviously simple and inexpensive, while the second is more accurate.^(^ ^8^ ^)^ This study was designed to determine whether total lymphocyte and CD3^+^ T cell counts peripheral blood are equivalent ATG dose monitoring strategies.

## OBJECTIVE

To investigate correlations between total lymphocyte and CD3^+^ T cell counts in the peripheral blood of renal transplant recipients treated with anti-thymocyte globulin, and describe related outcomes.

## METHODS

Retrospective study approved by the Ethics Committee of *Hospital de Clínicas de Porto Alegre,* official opinion number 1.152.441. This project was also approved at *Plataforma Brasil,* CAAE: 42923714.9.0000.5327 and was exempt from obtaining Informed Consent Terms.

A total of 667 isolated kidney transplants were performed in adult recipients at our organization between January 2008 and December 2013. Of these, 526 (78.9%) involved organs obtained from deceased donors and 141 (21.1%) from living donors. Indications for ATG-based induction therapy included immunological risk (panel reactive antibodies - PRA >50); presence of donor-specific antibodies (DSA) determined via Luminex^®^ platform; donor with acute renal failure and terminal serum creatinine ≥2.0mg/dL; and cold ischemia time (CIT) >24 hours. Indications for therapeutic use of ATG were steroid-resistant rejection and acute cellular rejection ≥ Banff 2A. A minimum of two doses of polyclonal T-cell depleting antibodies were used for induction or treatment of severe acute rejection in 201 (30.1%) and 25 (3.7%) patients, respectively.

Patients who died within 24 hours of transplantation, receiving less than two doses of ATG and aged under 18 years were excluded from analysis.

Patients with high immunological risk were submitted to ATG-based induction therapy consisting of four doses of 1.5mg/kg. Otherwise, ATG was given to achieve serum creatinine levels of 2.5mg/dL or up to four doses, whichever occurred first. Tacrolimus was initiated on the day of the last ATG dose.

The first dose of ATG was given intraoperatively and subsequent ones whenever CD3^+^ T cell counts increased to >20 cells/mm^3^. In rejection cases, ATG was given at 1.5mg/kg, then according to CD3^+^T counts for 7 to 14 days, depending on rejection severity and graft response to treatment. Leukocytes and CD3^+^ T cells were counted daily.

All recipients were treated with corticosteroids, sodium mycophenolate and a calcineurin inhibitor (predominantly tacrolimus). Anti-thymocyte globulin immune modulating effects were monitored via CD3^+^ T cell and/or total lymphocyte counts in peripheral blood.

The absolute number of peripheral lymphocytes was determined via automated white blood cell count (Sysmex XE-5000, Sysmex Corporation, Japan) carried out according to manufacturer's instructions. Peripheral CD3^+^ T cells were counted by flow cytometry (FACSCanto™ II, BD Bioscience, USA) using anti-CD45 (anti-CD45 FITC-conjugated, BD Bioscience, Beckman Coulter, Exbio, Inc.) and anti-CD3 (anti-CD3 PE-conjugated, BD Bioscience, Beckman Coulter, Exbio, Inc.) monoclonal antibodies.

Approximately 4mL of peripheral blood were collected into EDTA-coated tubes; cells were then labelled with abovementioned antibodies, submitted to flow cytometry (acquisition of up to 200.000 events) and analyzed using Infinicyt™ software (Cytognos SL, Spain). CD3^+^ T lymphocytes were identified by the CD45^hi^/CD3^+^ phenotype and the percentage of CD3^+^ T cells estimated from leukocyte (CD45^+^ cells) counts.

### Statistical analysis

Descriptive analysis of frequency and demographics are presented as mean ± standard deviation, medians or percentages. Statistical analysis was performed using SAS, version 9.4 and Statistical Package of the Social Science (SPSS), version 18, software. Data were analyzed using the Spearman's correlation and Kappa coefficients. A Receiver Operator Characteristics (ROC) curve was generated for analysis of diagnostic parameters. Survival curves were obtained via the Kaplan-Meier method. The level of significance was set at 5% (p<0.05).

## RESULTS

Demographic and transplant-related characteristics are shown in table 1. Anti-thymocyte globulin was used prophylactically or to treat acute rejection in 201 and 25 kidney graft recipients, respectively. Recipient characteristics were as follows: middle-age, equal distribution of males and females, predominantly of Caucasian origin and receiving grafts from deceased donors. Anti-thymocyte globulin was given prophylactically to 158 patients (78.6%) with high immunologic risks (high PRA, presence of DSA or positive flow cytometry cross-match), 40 patients (19.9%) with CIT >24 hours, and 3 recipients (1.5%) of grafts obtained from donors with acute renal failure. Therapeutic ATG administration was limited 13 patients (52%) with Banff ≥2A and 12 patients (48%) with steroid resistant rejection.

**Table 1:** Demographic characteristics of recipients and donors, and transplant-related variables

Characteristics and variables		Results
Recipient characteristics		
Age, years		46.3±13.5
Male		115 (50.9)
Ethnicity		
	White/not white	179 (79.2)/47 (20.8)
Primary kidney disease		
	Hypertension	55 (24.3)
	*Diabetes mellitus*	25 (11.1)
	APKD	22 (9.8)
	Glomerulonephritis	20 (8.9)
	Obstructive uropathy and reflux	8(3.5)
	Others	8(3.5)
	Unknown	88 (38.9)
Donor characteristics		
Donor		
	Deceased	192(85)
	Living	34 (15)
Donor age, years		42.6±17.3
ECD		83 (36.7)
HLA-ABDR mismatches		3.1±1.2
Transplant-related variables		
	Peak class I PRA	23.5(0-67)
	Peak class II PRA	18.5 (0-50.3)
	Last class I PRA	11.0 (0-55)
	Last class II PRA	5 (0-35.3)
Living donor compatibility		
	HLA identical	1 (2.9)
	1 haplotype	29 (85.3)
	0 haplotype	3(8.9)
	Unrelated (spouse)	1 (2.9)

Results expressed as mean±standard deviation, n (%) and median (percentile 25-75).

APKD: adult polycystic kidney disease; ECD: expanded criteria donors; HLA: human leukocyte antigens; ABDR: human leukocyte antigens class I (A and B) and class II (DR); PRA: panel reactive antibodies.

Mean prophylactic or therapeutic ATG cumulative doses amounted to 5.6±1.3mg/kg and 7.2±3.4mg/kg, respectively; the mean cumulative dose per patient corresponded to 5.8±1.7mg/kg. The number of ATG doses given per patient was as follows: 7 patients (3.1%), 2 doses; 52 patients (23%), 3 doses; 113 (50%), 4 doses; and 54 patients (23.9%), 5 or more doses.

**Figure 1:**
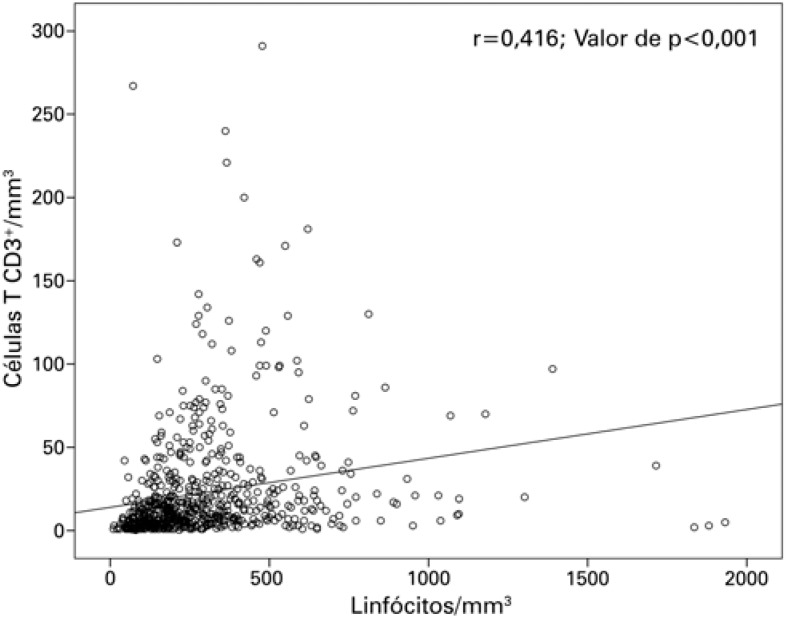
Correlation between CD3^+^ T cells and total lymphocyte cell counts

Paired total lymphocyte and CD3^+^ T cell counts were available for 664 samples obtained from 226 kidney transplant recipients receiving ATG for induction or treatment of acute rejection. Correlations between total lymphocyte and CD3^+^ T cell counts are illustrated in figure 1. Spearman's correlation coefficients corresponded to 0.41 (all samples combined; p<0.001), 0.43 (samples obtained from patients receiving prophylactic ATG; p<0.001), and 0.28 (samples obtained from patients receiving therapeutic ATG; p<0.005). The overall Kappa coefficient was 0.27 (p<0.001); Kappa coefficients of 0.28 (p<0.001) and 0.15 (p=0.081) were calculated for samples obtained from patients receiving prophylactic and therapeutic ATG, respectively. The best cut-off for lymphocytes numbers (ROC curve) was 256, with sensibility of 66.8% and specificity of 66.9% (AUC: 0.71; 95% confidence interval – 95%CI 0.67-0.75). Increased sensitivity and negative predictive values were obtained by reducing the number of peripheral lymphocytes that would correlate with a number of CD3^+^ T cells <20 cells/mm^3^. However, this was associated with a significant drop in accuracy (Table 2).

**Table 2:** Diagnostics parameters according to total lymphocyte counts in peripheral blood

Lymphocyte cutoff	Sensitivity (%)	Specificity (%)	PPV (%)	NPV (%)	Accuracy (%)
300	55.4	73.4	47.7	79.0	67.9
256	66.8	66.5	46.6	82.1	66.6
200	79.7	53.5	42.8	85.8	61.4
150	89.6	37.0	38.3	89.1	53.0
100	98.0	19.7	34.8	95.8	43.5

PPV: positive predictive value; NPV: negative predictive value.

None of the patient was diagnosed with acute rejection while receiving prophylactic ATG. However, 39 (19.4%) patients developed acute rejection after ATG was discontinued. Of these, 23 were cellular rejections, 14 antibody-mediated rejections and 2 mixed rejections. Ten patients suffered a second rejection episode and two patients a third. In all patients presenting with repeated rejections, ATG had been given due to high immunological risks.

**Figure 2:**
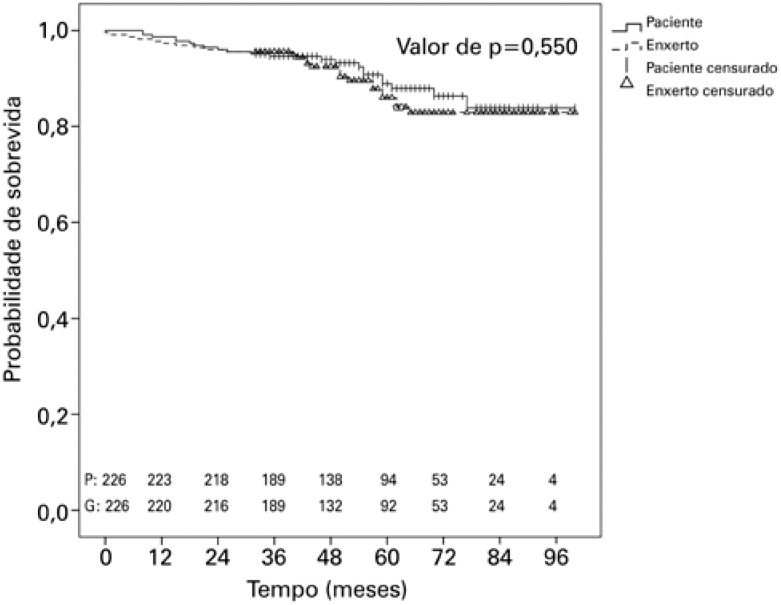
Estimated (Kaplan-Meier) overall patient and graft survival P: patient; G: graft.

**Figure 3:**
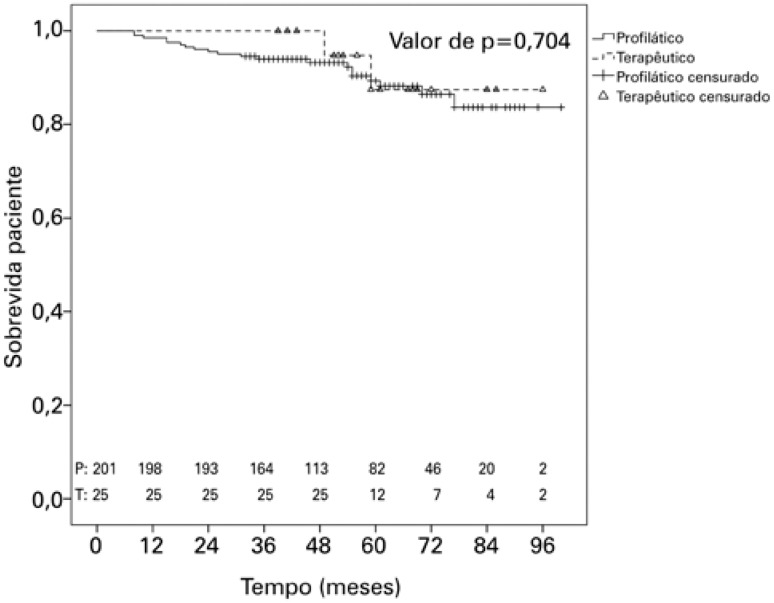
Estimated (Kaplan-Meier) patient survival according to anti-thymocyte globulin indication P: prophylactic; T: therapeutic.

**Figure 4:**
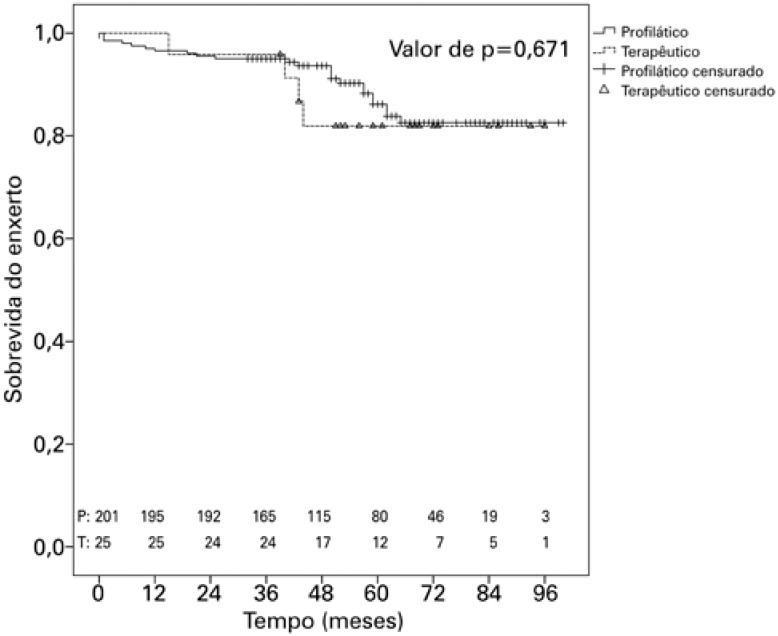
Estimated (Kaplan-Meier) graft survival according to anti-thymocyte globulin indication P: prophylactic; T: therapeutic.

Overall patient and graft survival is shown in figure 2. One- and five-year patient and graft survival rates corresponded to 98.7% and 97.8%, and 88.8% and 87.4%, respectively. Patient survival according to ATG indication is shown in figure 3. Patient survival rates within 1 and 5 years of transplant corresponded to 98.5% and 100% (prophylactic ATG group), and 89.2% and 87.4% (therapeutic ATG group). Graft survival according to ATG indication is displayed in figure 4. One- and 5-year graft survival rates were 97.5% and 100%, and 88.2% and 81.7% for prophylactic and therapeutic ATG therapy, respectively. Functional graft parameters according to ATG indication within 6 and 12 months of transplantation are shown in table 3. Mildly improved according to Chronic Kidney Disease Epidemiology Collaboration (CKD-EPI) estimated glomerular filtration rates (eGFR) were documented at both time points, regardless of ATG indication. In patients with acute rejection, median baseline (start of ATG therapy) serum creatinine concentration was 2.9mg/dL (P25-75: 2.0-6.3), with peak values of 3.4mg/dL (P25-75: 2.5-7.4) during treatment and drop to 2.4mg/dL (P25-75: 1.7-4.0) at the end of the ATG course.

**Table 3:** Renal graft function parameters according to anti-thymocyte globulin indication

		Results	
		6 months	12 months
Prophylactic ATG (n=201)			
	Serum creatinine	1.4 (1.2-2.0)	1.4 (1.1-1.8)
	Estimated CKD-EPI GFR	49.7±23.7	54.3±24.6
	PCR	1.4 (1.2-2.0)	1.4 (1.1-1.8)
Therapeutic ATG (n=25)		
	Serum creatinine	1.6 (1.3-2.2)	1.6 (1.1-2.6)
	Estimated CKD-EPI GFR	45.4±17.4	47.4±21.3
	PCR	0.15 (0.1-0.5)	0.2 (0.1-0.5)

Results expressed as median (percentil 25-75) and mean±standard deviation.

GFR: glomerular filtration rate; CKD-EPI: Chronic Kidney Disease Epidemiology Collaboration; PCR: protein-creatinine ratio; ATG: anti-thymocyte globulin.

## DISCUSSION

This retrospective non-controlled cohort study set out to investigate correlations between CD3^+^ T cell and total lymphocyte counts in anti-T cell polyclonal antibody dose monitoring. Different from flow cytometry, which is able to accurately determine T cell numbers, lymphocyte count in peripheral blood does not discriminate between B, T, natural killers (NK) and other innate lymphoid cells. Therefore, we hypothesized flow cytometry would be a superior monitoring method compared to lymphocyte count in peripheral blood.

Polyclonal anti-T cell antibodies have been used in transplant recipients for decades, either as part of induction regimens or for treatment of acute rejection episodes.^(^ ^9^ ^)^ Induction therapy is aimed to suppress cellular and humoral responses and prevent acute rejection. Rabbit ATG, interleukin (IL) 2 receptor blockers, and anti-CD-52 monoclonal antibodies are the most common antibodies used in organ transplant recipients for induction purposes. Roughly 80% of transplant centers in the United States currently employ antibody induction therapy, especially with polyclonal anti-T cell antibodies.^(^ ^10^ ^)^ These consist of xenoproteins produced in response to injection of human thymocytes, lymphoblasts or lymphocytes into animals, particularly rabbits, from which purified immunoglobulins are then obtained.^(^ ^11^ ^)^ Polyclonal anti-T cell antibodies can be used as a general strategy in nearly all patients, or given in an individualized manner to those with high immunological risks or prolonged CIT. In patients receiving kidneys from donors with acute injuries, these products can be used to avoid early administration of calcineurin inhibitors.^(^ ^12^ ^)^ These agents can also be used in acute graft rejection, either as primary treatment or rescue therapy for steroid-resistant rejections.^(^ ^13^ ^)^ Significant benefits derived from the use of ATG in renal transplantation include low rejection rates, enhanced graft survival and function, potentially facilitated development of regulatory T cells implicated in suppression mechanisms, such as CD4^+^/CD25^+^/ FOXP3^+^, and improved graft survival.^(^ ^14^ ^)^ According to a recent comprehensive analysis, antibody induction seems to be highly beneficial in kidney transplant, both from a cost and an outcome perspective, ATG being the most effective agent.^(^ ^15^ ^)^ However, these products may induce excessive immunosuppression, with increased risks of opportunistic infections and neoplastic disorders.^(^ ^16^ ^,^ ^17^ ^)^

With ATG indications and biological effects in mind, strategies aimed to decrease patient exposure while maintaining treatment efficacy have been developed to replace fixed dose regimens. Several protocols have been proposed according to biological effects of ATG on T cells.^(^ ^18^ ^–^ ^20^ ^)^ This type of strategy was introduced some decades ago by Cosimi et al., as a means to improve treatment efficacy, and became the standard of care ever since.^(^ ^21^ ^)^ At this stage, CD3^+^ T cell count in peripheral blood using flow cytometry is the monitoring method of choice.^(^ ^22^ ^,^ ^23^ ^)^ Nevertheless, this tool is unavailable in many transplant centers, which must rely on lymphocyte counts in peripheral blood to monitor the effects of polyclonal anti T-cell antibody therapy.^(^ ^24^ ^)^

Previous studies investigating correlations between CD3^+^ T cell and total lymphocyte counts in peripheral blood have been published.^(^ ^25^ ^,^ ^26^ ^)^ Cut-offs for CD3^+^ T cell and total lymphocyte counts employed in these correlation studies varied, but most adopted cut-offs of 20 CD3^+^ T cells/mm^3^ (flow cytometry) and 200 peripheral lymphocytes/mm^3^.^(^ ^27^ ^)^

In a retrospective analysis of cellular graft rejection in 302 patients, rejection was associated with >20 CD3^+^ T cells/mm^3^ in 91 cases.^(^ ^28^ ^)^ In that same study, peripheral blood lymphocyte counts <200 cells/mm3 and CD3^+^ T cell counts <20 cells/mm3 were found to be highly correlated in a small sample of patients. Franco et al.,^(^ ^29^ ^)^ evaluated 298 samples obtained from 24 kidney transplant recipients receiving ATG for induction or treatment of acute rejection. Cut-off values of 10 CD3^+^ T cells/mm^3^ and <100 peripheral blood lymphocytes/ mm^3^ were used in that study, with significant correlations limited to days 5 and 15 of ATG administration. Significant differences between methods were therefore reported.^(^ ^29^ ^)^ Ata et al., analyzed two groups of renal transplant patients undergoing ATG-based induction therapy and monitored via total lymphocyte or CD3^+^ T cell counts. Higher immunomodulation instability requiring higher doses of ATG and implying higher costs was observed in the first group. Rejection and infection rates did not differ significantly between groups in the first three months post-transplantation. It was argued that ATG monitoring based on CD3^+^ T cell counts is cost-effective, and that total lymphocyte counts are not correlated with CD3^+^ T cell counts.^(^ ^30^ ^)^

This study comprised 226 kidney transplant recipients treated with polyclonal anti-T cell antibodies for preventive or therapeutic purposes. In this cohort, 664 paired samples were submitted to flow cytometry and lymphocyte count in peripheral blood. Correlations were weak and indices of agreement low, in spite of statistical significance. Worthy of notice, when CD3^+^ T cell counts were kept below 20 cells/mm3 (standard of care), parameters obtained using the cut-off of 200 cells/mm^3^ in peripheral blood were marked by low specificity and positive predictive values, meaning that many patients would have been misclassified in terms of CD3^+^ T cell modulation. The best cut-off (ROC curve) achieved 66% sensitivity and specificity. Lower lymphocyte cut-off values increase sensitivity and negative predictive value at a substantial loss of specificity and positive predictive value. Increased falsepositive rates may lead to unnecessarily administration of ATG, augmenting the risks of significant adverse effects.

Results of this study suggest that total peripheral lymphocyte depletion may not be a good ATG therapy monitoring strategy, and should be replaced by flow cytometric CD3^+^ T cell count. If flow citometry is unavailable, clinicians must weight infection/rejection risks against available alternatives, *i.e.,* implementing ATG-based therapy in modulated patients based on lower lymphocyte count cut-offs, or not giving it to non-modulated patients based on higher cut-offs.

This work has some limitations, such as retrospective design, single center involvement, low number of samples obtained from patients undergoing rejection therapy and absence of baseline CD3^+^ T cell counts obtained prior to anti-T cell antibody administration. However, we believe findings to be of relevance for clinical practice. Also, we suggest CD3 ^+^ T cell counting by flow cytometry should be adopted as the gold standard to monitor polyclonal anti-T cell antibody administration.

## CONCLUSION

Results of this study suggest lymphocyte count in peripheral blood is not equivalent to CD3^+^ T cell count determined by flow cytometry as far as anti-T cell polyclonal antibody therapy monitoring, and dose adjustment is concerned, regardless of indication (*i.e*., prevention or treatment of acute kidney allograft rejection).
